# The bioinformatic approach identifies *PARM1* as a new potential prognostic factor in osteosarcoma

**DOI:** 10.3389/fonc.2022.1059547

**Published:** 2023-03-06

**Authors:** Haijun Feng, Liping Wang, Jie Liu, Shengbao Wang

**Affiliations:** ^1^ Department of Orthopedics, Second Hospital of Lanzhou University, Lanzhou, Gansu, China; ^2^ Department of Neurosurgery, Liaocheng Second People’s Hospital, Liaocheng, Shandong, China; ^3^ Second Hospital of Lanzhou University, Lanzhou, Gansu, China

**Keywords:** osteosarcoma, prognosis, PARM1, bioinformatics, GEO

## Abstract

**Objective:**

To explore the key factors affecting the prognosis of osteosarcoma patients.

**Methods:**

Based on the GEO dataset and differential expression analysis of normal and osteosarcoma tissues, the gene modules related to the prognosis of osteosarcoma patients were screened by WGCNA, and intersecting genes were taken with differential genes, and the risk prognosis model of osteosarcoma patients was constructed by LASSO regression analysis of intersecting genes, and the prognosis-related factors of osteosarcoma patients were obtained by survival analysis, followed by target for validation, and finally, the expression of prognostic factors and their effects on osteosarcoma cell migration were verified by cellular assays and lentiviral transfection experiments.

**Results:**

The prognosis-related gene module of osteosarcoma patients were intersected with differential genes to obtain a total of 9 common genes. PARM1 was found to be a prognostic factor in osteosarcoma patients by LASSO regression analysis, followed by cellular assays to verify that PARM1 was lowly expressed in osteosarcoma cells and that overexpression of PARM1 in osteosarcoma cells inhibited cell migration. Pan-cancer analysis showed that PARM1 was lowly expressed in most cancers and that low expression of PARM1 predicted poor prognosis for patients.

**Conclusion:**

The data from this study suggest that PARM1 is closely associated with the prognosis of osteosarcoma patients, and PARM1 may serve as a novel potential prognostic target for osteosarcoma, providing a heartfelt direction for the prevention and treatment of osteosarcoma.

## 1 Introduction

Osteosarcoma is common primary malignant bone tumors of mesenchymal (1, 2). Osteosarcoma occurs in the blood-rich epiphyses of long bones of the extremities, such as the distal femur and proximal tibia. The clinical symptoms of osteosarcoma are usually local swelling and pain, occasionally accompanied by joint mobility disorders, and it has a high tendency to metastasize, and is highly susceptible to early metastases, especially pulmonary metastases, and the disease progresses rapidly (3, 4). The treatment of osteosarcoma has evolved from early surgical amputation alone to comprehensive treatment with neoadjuvant chemotherapy combined with limb-preserving surgery (5, 6). The 5-year survival rate of patients with limited osteosarcoma has improved from 20% to 60%-70% with this combined treatment modality, but further improvement in treatment outcomes is bottlenecked at this stage, while the survival rate of patients with metastatic and recurrent osteosarcoma remains low (7–9). Therefore, it is still urgent to deeply investigate the complex cellular mechanisms and molecular signaling mechanisms of pathogenesis and progression in osteosarcoma.

With the development of technology in recent years, high-throughput sequencing technology has been widely used and is an important tool for diseases such as cancer. The GEO database contains a large amount of oncogene expression data (10–12). For example, Guo et al. (13) identified pivotal genes and pathways in a rat model of renal ischemia-reperfusion injury based on bioinformatics analysis of GEO microarray datasets and integration of gene expression profiles. Ma et al. (14) screened for differential mRNA expression between brain tissue and blood by mining Alzheimer’s patient data in the GEO dataset and found that ITGB1 and RAB7A could be used as biomarkers for Alzheimer’s disease. In addition, Li et al. (15) identified *MMP9, CD74, SPP1, CXCL12, TYROBP, FCER1G* et al. as central genes for osteosarcoma initiation through the GEO database.

To screen for differentially expressed genes in osteosarcoma cells compared to normal cells, find the biological role of differential genes in cancer cells, verify the correlation between differential genes and survival prognosis of osteosarcoma patients, and provide a theoretical basis for identifying targets for clinical osteosarcoma treatment.

## 2 Methods

### 2.1 Data source

All the data in this study come from the target database (https://ocg.cancer.gov/programs/target), and the GEO database (https://www.ncbi.nlm.nih.gov/geo/), the target database contains the mRNA expression profile and clinical data of 98 osteosarcoma patients were obtained from the GEO database GSE16088 and GSE16091 data sets. The mRNA expression profile data and clinical data of 6 normal controls and 48 osteosarcoma patients were obtained. Normalize and debatch the obtained GEO data, standardize the samples, and view the clustering of the sample groups through the PCA graph and the UMAP graph.

### 2.2 Expression difference analysis

The DESeq2 package of R software was used for expression profile difference analysis as well as gene volcano and heat map plotting. Corrected P values and |log2FC| were calculated, with P<0.05 and |log2FC|≥1.0 for differentially expressed genes. The Gene Ontology (GO) analysis was visualized using the ClusterProfiler package in R software, and a false discovery rate <0.01 was statistically significant.

### 2.3 WGCNA

The GSE16091 dataset included expression profiles, and survival events of 34 osteosarcoma tissues, which were suitable for the construction of weighted gene co-expression networks. GSE160306 and GSE160308 gene expression data matrices were constructed using the R package “ WGCNA”, and the top 25% of genes with the largest differences in the samples were selected as the input dataset for the subsequent WGCNA. A sample hierarchical clustering approach was used to detect and remove abnormal samples before selecting the appropriate soft threshold function. The neighbor-joining matrix and topological overlap matrix were constructed to calculate the corresponding dissimilarities and complete the identification of gene trees and modules. The minimum module size was 20. then the highly similar modules were merged by clustering and fusing module feature genes. The degree of difference is less than 0.25. Average linkage hierarchical clustering was established, similar genes were divided into a module, and osteosarcoma grading was used as phenotype data to screen the optimal module.

### 2.4 Survival analysis

The impact of prognostic survival of osteosarcoma was assessed by overall survival (OS). All survival analyses were performed using COX multiple survival regression model analysis as the primary method and the Kaplan-Meier method as an adjunct. The endpoint of overall survival was defined as the time from the time of randomization to death from any cause, and all methods and R packages were performed using R software version v4.0.3. p < 0.05 was statistically significant.

### 2.5 Prognostic feature models

The collection between gene expression and OS was assessed using LASSO COX analysis. A prognostic risk prediction model for osteosarcoma was built. Patients with osteosarcoma were divided into high and low-risk groups based on the median risk score. KM curves were plotted to compare OS between the high-risk and low-risk parts, and ROC survival analysis was performed using the R package SURVIVAL, and the “rmda” package was used for decision curve analysis.

### 2.6 Cell culture and transfection

The derived human osteoblast cell line hfob 1.19 and osteosarcoma cell line saos-2 were cultured in a 1:1 mixture of Ham’s F12 medium and Dulbecco’s modified Eagle’s medium containing 2.5 mM L -gu Aminoamide (ATCC, LGS standard, PL), supplemented with 100 U/mL penicillin, 100 U/mL streptomycin (Sigma-Aldrich, Warsaw, Poland), 0.3 mg/mL G418 (Sigma-Aldrich, Warsaw, Poland) and 10% fetal bovine serum (v/v, FBS, Gibco, Thermo Fisher Scientific, Warsaw, Poland). Cells were grown at 34°C, 5% CO_2_. The pc DNA3.1 transfection plasmid was purchased from Genechem Co., Ltd. (Shanghai, China). Osaos-2 cells were seeded in 6-well plates (2×10^5^/well), and after incubation for 24 hours, they were transfected with LiPofectamine 2000 (corresponding plasmid 100 nM), and the operation was performed according to the instructions. Subsequent experiments were performed 48 hours after transfection.

### 2.7 Quantitative reverse transcription PCR (RT-qPCR)

Total RNA was extracted from each group of cells using TRIzol reagent (Invitrogen). cDNA was synthesized from RNA using a Primescript RT kit (Takara, Japan). The cDNA was amplified and quantified using SYBR Green mix (Finnish Finnzymes) in an Applied Biosystems 7500 instrument. The primer sequences used are shown in **Table 1**. -2^ΔΔCt^ method could determine the gene expression.

**Table 1:** Primer sequences.

Name	sequence
*IFIT1*-F	TCAGAAGTCTAGTCACTTGGGG
*IFIT1*-R	ACACCTTCGCCCTTTCATTTC
*PARM1*-F	TCTTAACTGCAGGATGGAGGG
*PARM1*-R	TAGTCCAGATGGTGGTCGGT
GAPDH-F	TTGATTTTGGAGGGATCTCGCTC
GAPDH-R	GAGTCAACGGATTTGGTCGTATTG

### 2.8 Western Blot (WB)

Cells were collected, lysed in lysate (phosphatase inhibitor, protease inhibitor and PMSF), and protein quantification was performed by BCA method (Thermo Fisher Scientific, China). 10-20 μg protein was loaded on 8%-12% 30% acrylamide-Bis gel, then transferred through 0.22 μm pore PVDF membrane (Merck Millipore, USA), and blocked with 5% nonfat milk powder for 1 h., respectively, add primary antibodies (Rabbit polyclonal to IFIT1, 1:1000, ab236256; Rabbit monoclonal [EPR10009] to PARM-1, 1:1000, ab168369, abcam) and incubate overnight at 4°C. The next day, secondary antibodies (Goat Anti-Rabbit actin, 1:2000, ab8226, abcam) were added and incubated at room temperature for 1 h, and then luminescent solution (Thermo Fisher Scientific, China) was added for exposure and color development. Image J software was used for analysis, and the relative protein content was expressed as the gray value of the corresponding protein band/the gray value of the actin protein band, which was repeated three times.

### 2.9 Transwell

Digest the cells and adjust to 1×106 cells/mL in culture medium without serum. Matrigel (BD Biosciences, San Jose, CA, USA) was added to the upper chamber and incubated at 37°C for 2 h. Add 100 µL of cell suspension to the upper chamber and 600 µL of cell culture medium with FBS to the bottom chamber. Subsequently, the cells were incubated at 37°Cand 5% CO_2_ in an ambient environment for 24 hours. Detach the upper chamber, remove the cells, and fix with 4% paraformaldehyde. Cells were washed with phosphate-buffered saline (PBS) and stained with crystal violet. Stained cells were observed with a microscope (Olympus, Tokyo, Japan).

### 2.10 Statistical analysis of data

SPSS 24.0 and GraphPad Prism 8.0.1 software can process the data. Normally distributed measures are expressed as (x ± s), and comparisons between two parts were made by independent samples t-test, and P was a two-sided test, and differences were statistically significant at P < 0.05.

## 3 Results

### 3.1 Differential gene screening

**Figure 1:**
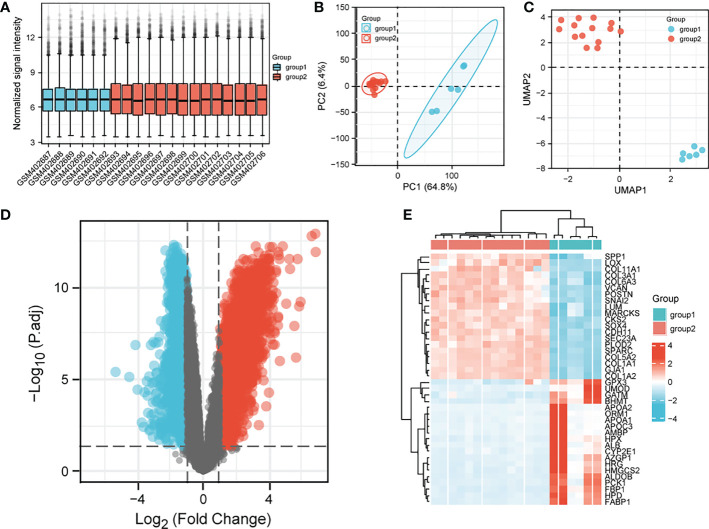
Analysis of differential genes in the GSE16088 dataset. **(A)** sample normalized box plot, vertical coordinates indicate sample normalized intensity; **(B)** sample PCA plot; **(C)** UMAP clustering plot; **(D)** volcano plot; **(E)** heat map; Top20 differential genes are shown. Normal in the figure indicates normal tissue and the OS indicates osteosarcoma tissue.

We downloaded the dataset of GSE16088 from the GEO database, which included 14 cases of osteosarcoma tissues and 6 cases of normal tissues, first, we analyzed the datasets, which showed good normalization and differences between samples (**Figures 1A–C**). We then analyzed the differentially expressed genes in normal and osteosarcoma tissues, and there were 5005 differentially expressed genes (2719 significantly up-regulated genes, 2286 down-regulated genes), **Figures 1D, E** show the differential gene volcano and heat map.

### 3.2 WGCNA identification of genes associated with osteosarcoma survival orientation

**Figure 2:**
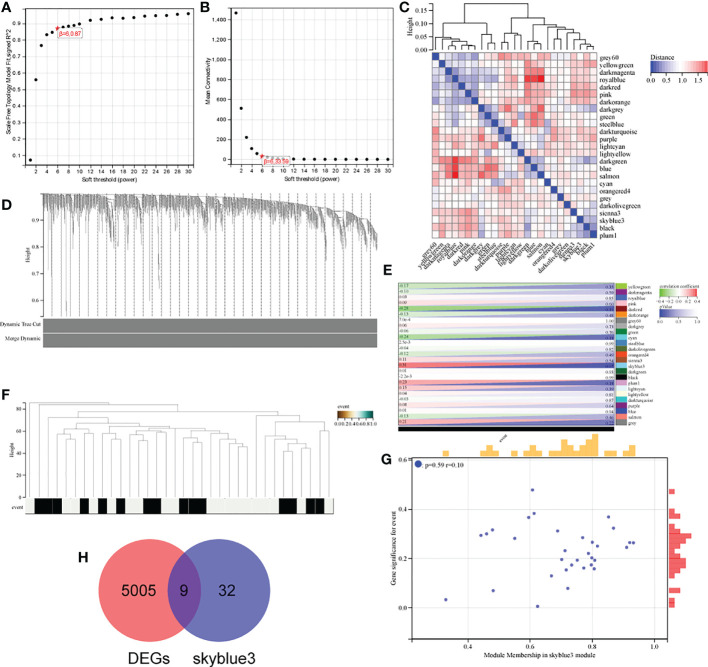
Identification of genes associated with osteosarcoma survival orientation. **(A, B)** analysis of the average connectivity of the scale-free fit index and various soft threshold functions. Assessment of scale-free topology at β = 6; **(C)** Gene module distribution mapping; **(D)** Gene vs. sample clustering map; **(E)** Heat map of module feature genes associated with individual osteosarcoma survival; **(F)** Tree and clinical feature heat map; **(G)** Scatter plot of skyblue3 module genes; **(H)** Venn diagram of skyblue3 module genes and DEGs.

To identify genes associated with osteosarcoma survival orientation, we constructed co-expression networks by WGCNA in GSE16091, and in this study, we chose β=6 as the threshold to construct scaling-free networks (**Figures 2A, B**), and a total of 25 gene expression modules were identified after using merged dynamic tree cuts (**Figure 2C**), by constructing a random gene network map (**Figure 2D**). By calculating the correlation between module feature genes and clinical features, the skyblue3 module was most strongly correlated with the survival of osteosarcoma patients (**Figure 2E**), and the neighbor-joining matrix was clustered with the clinical features of the samples in **Figure 2F**, and the scatter plot of skyblue3 module genes in **Figure 2G**. We then obtained a total of 9 genes (*FI27 ISG15, IFI6, IFI44L, PARM1, MX1, IFIH1, IFIT1, RSAD2*) by taking the intersection of skyblue3 module genes and DEGs **Figure 2H**.

### 3.3 Prognostic risk construction based on intersecting genes

**Figure 3:**
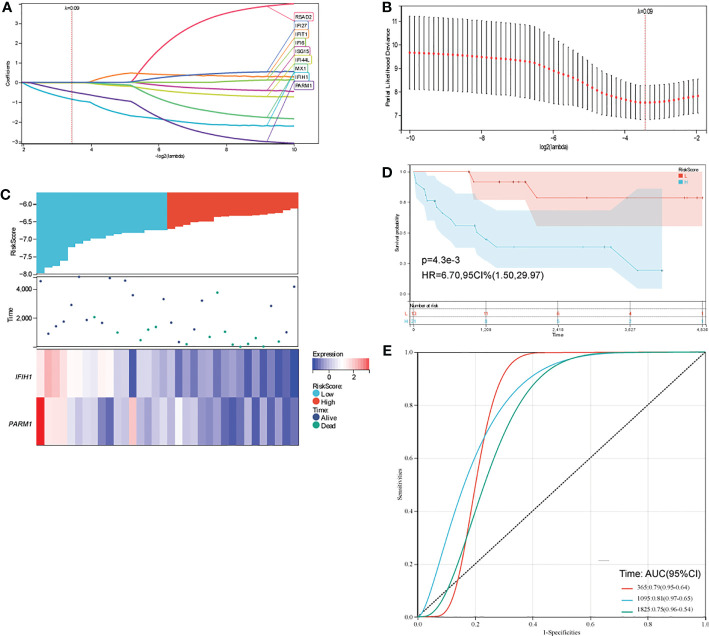
**(A, B)** distribution of LASSO coefficients for key genes to obtain the adjustment parameter l.min= 0.09, and the vertical black dashed line in B defines the optimal l value; **(C)** Riskscore distribution, survival status and duration, and gene expression of prognostic characteristics of osteosarcoma patients are shown from top to bottom; **(D)** KM survival curves for high and low-risk groups; **(E)** ROC curves at 1, 3 and 5 years.

To explore the prognostic impact of intersecting genes of modular genes and DEGs on osteosarcoma, we did prognostic models based on LASSO analysis for these nine genes, obtained the value of the independent variable lambda and the coefficient of the independent variable (**Figure 3A**), and plotted the partial likelihood deviation versus log(λ) (**Figure 3B**). Patients with osteosarcoma were subsequently divided into high and low-risk groups (**Figure 3C**) by the hazard score Riskscore=(-0.8635)* *IFIT1*+(-0.481)* *PARM1*, and their survival status was presented in **Figure 3C**. **Figure 3D** shows the difference in survival between the high and low-risk parts (p=4.3e-3). Prognostic survival was predicted for 1, 3, and 5 years, and this showed good sensitivity and specificity (**Figure 3E**).

### 3.4 Effect of *IFIT1* and *PARM1* on the prognosis of patients with osteosarcoma

**Figure 4:**
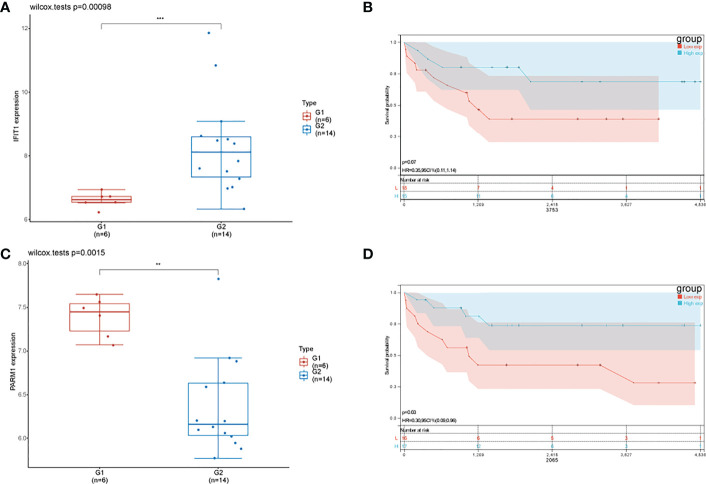
Effect of *IFIT1* and *PARM1* on the prognosis of osteosarcoma patients. **(A)** Expression of *IFIT1* in normal and osteosarcoma tissues in GEO dataset; **(B)** KM curve showing the effect of high and low expression of *IFIT1* on the prognosis of osteosarcoma patients; **(C)** Expression of *PARM1* in normal and osteosarcoma tissues in GEO dataset; **(D)** KM curve showing the effect of high and low expression of PARM1 on the prognosis of osteosarcoma patients; ** means p<0.01, *** means p<0.001.

In order to further investigate the role of *IFIT1* and *PARM1* in patients with osteosarcoma, we analyzed the expression and prognostic effects of *IFIT1* and *PARM1* in patients with osteosarcoma, using the risk scores of *IFIT1* and *PARM1*. *IFIT1* expression was significantly increased in osteosarcoma tissues compared to normal tissues (**Figure 4A**), and patients with low *IFIT1* expression had a better prognosis (**Figure 4B**, p=0.07). *PARM1* was lowly expressed in osteosarcoma tissues (**Figure 4C**), and low expression of *IFIT1* predicted a poor prognosis for patients (**Figure 4D**).

### 3.5 Cellular experiments to verify the expression of *IFIT1* and *PARM1*

**Figure 5:**
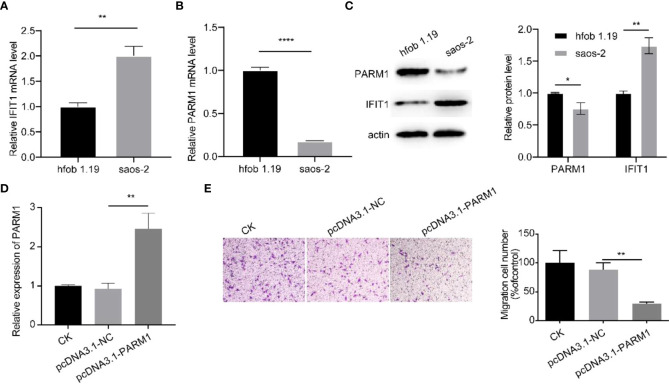
Cellular experiments to verify the expression of *IFIT1* and *PARM1*. **(A, B)**: RT-qPCR to detect the mRNA levels of *IFIT1* and *PARM1* in normal and osteosarcoma tissues. **(C)** WB detection of IFIT1 and *PARM1* protein levels in normal and osteosarcoma tissues. **(D)** RT-qPCR detection of mRAN levels of PARM1 in different groups; **(E)**: transwell detection of cell migration ability in different groups; ** means p<0.01, *** means p<0.001, **** means p<0.0001.

To further verify the heterogeneity of *IFIT1* and *PARM1* in normal and osteosarcoma tissues, we cultured the osteoblast cell line hfob 1.19 and the osteosarcoma cell line saos-2, and detected the expression of *IFIT1* and *PARM1* by RT-qPCR, and the results showed that compared with osteoblasts, osteosarcoma cells had high expression of *IFIT1* (**Figure 5A**), *PARM1* was lowly expressed (**Figure 5B**). Then we detected the protein levels of IFIT1 and *PARM1* in the osteoblast cell line hfob 1.19 and the osteosarcoma cell line saos-2 by WB. The results showed that the expression of *IFIT1* was high and the expression of *PARM1* was low in the osteosarcoma cell line saos-2 (**Figure 5C**). The above data show that *IFIT1* is highly expressed and *PARM1* is lowly expressed in osteosarcoma tissues. Subsequently, we overexpressed PARM1 in saos-2 cells, the transfection efficiency is shown in **Figure 5D**, and on this basis, we detected the cell migration ability. The results showed that overexpressing PARM1 in osteosarcoma cells significantly reduced the migration ability of cells (**Figure 5E**, p<0.01).

### 3.6 Target database verification

**Figure 6:**
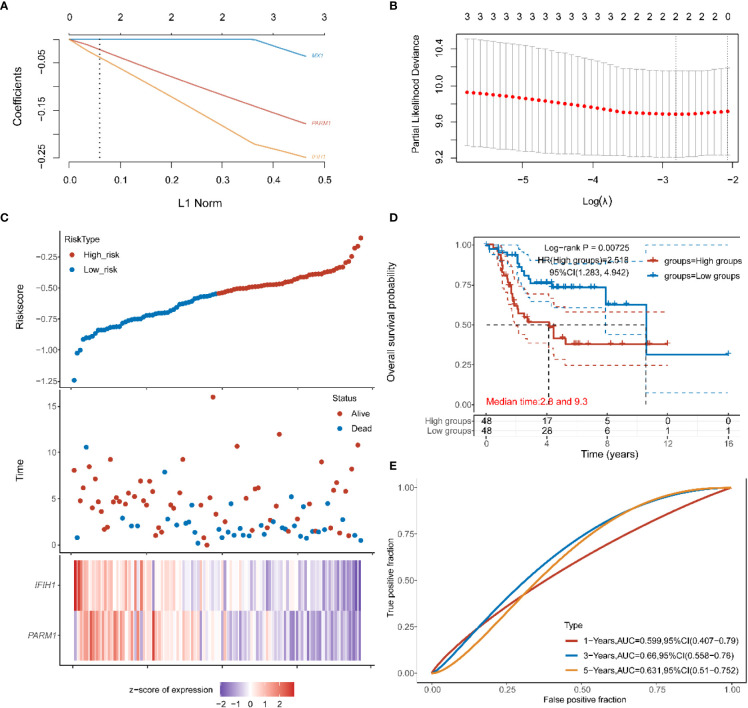
**(A, B)** in the target data: the distribution map of the LASSO coefficient of the key gene, and the adjusted parameter I.min= 0.0599, the vertical black dotted line in **(B)** defines the optimal I value; **(C)** Riskscore distribution, survival status and duration, and gene expression of prognostic characteristics of osteosarcoma patients are shown from top to bottom; **(D)** KM survival curves of high and low risk groups; **(E)** ROC curves of 1 year, 3 years and 5 years.

**Figure 7:**
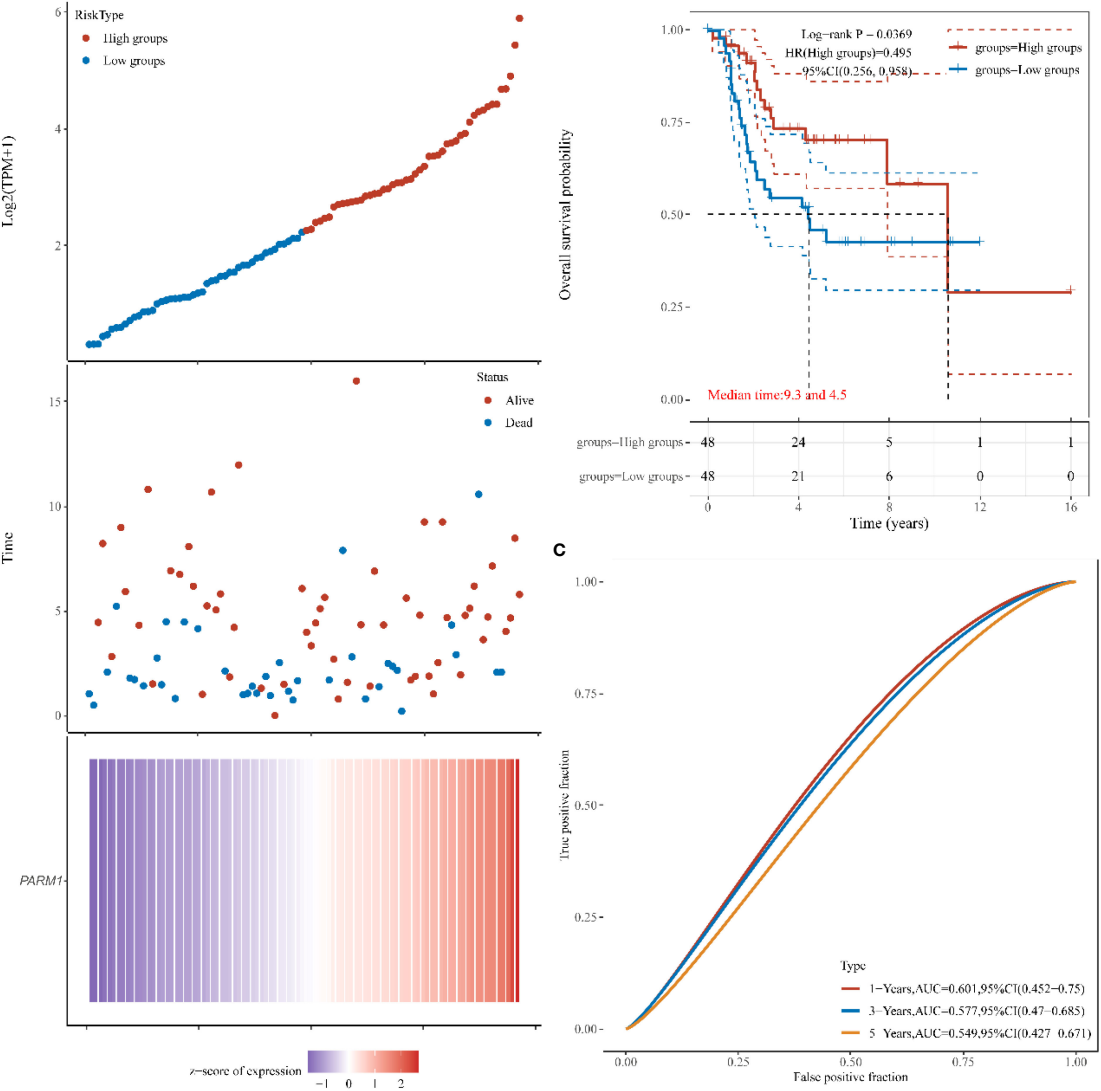
The effect of high or low expression of *PARM1* on the prognosis of osteosarcoma patients. **(A)**: *PARM1* expression distribution and survival distribution of osteosarcoma patients; **(B)**: *PARM1* high and low expression survival curve; **(C)**: ROC curves of 1 year, 3 years and 5 years.

In order to further verify the correctness of the constructed risk model, we constructed a prognostic risk model based on the target using 9 genes, *FI27 ISG15, IFI6, IFI44L, PARM1, MX1, IFIH1, IFIT1, RSAD2*, and obtained the independent variable lambda values ​​and the coefficients of the independent variable (**Figure 6A**), and plotted the partial likelihood deviation versus log(λ) (**Figure 6B**). Subsequently, patients with osteosarcoma were divided into high-risk group and low-risk group by the risk score Riskscore=(-0.0921)*PARM1+(-0.1418)*IFIH1 (**Figure 6C**), and their survival status was shown in **Figure 3C**. **Figure 6D** shows the difference in survival between high and low risk groups (P=0.00725). Prognostic survival was predicted for 1, 3, and 5 years, and the prognostic model showed good sensitivity and specificity (**Figure 6E**). Subsequently, we analyzed the effect of high or low expression of PARM1 on the prognosis of patients with osteosarcoma in the target database. **Figure 7A** shows the distribution of patients with osteosarcoma. The KM curve shows that patients with high PARM1 expression have a better prognosis (**Figure 7B**), and the ROC curve shows that patients with osteosarcoma have a better prognosis. Good sensitivity (**Figure 7C**).

### 3.7 *PARM1* pan-cancer expression and prognostic analysis

**Figure 8:**
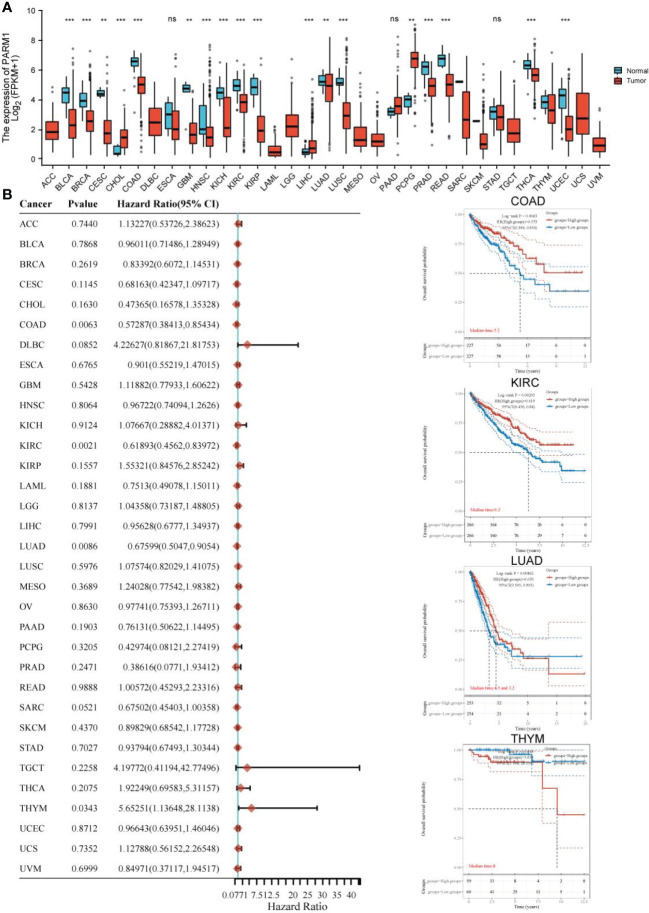
*PARM1* pan-cancer expression and prognosis analysis. **(A)** Expression of *PARM1* in cancer; **(B)** Effect of PARM1 expression level on cancer prognosis; ** means p<0.01, *** means p<0.001.

In the previous data, we confirmed that low expression of *PARM1* predicted poor prognosis in osteosarcoma patients. To further elaborate on the role of *PARM1* in cancer, we performed a pan-cancer expression and prognosis analysis of *PARM1*. As shown in **Figure 8A**, *PARM1* was lowly expressed in BLCA, BRCA, CESC, COAD, GBM, HNSC, KICH, KIRC, KIRP, LUAD, LUSC, PRAD, PEAD, THCA, and UCEC, and highly expressed in CHOL and LIHC. In addition, the expression level of *PARM1* was closely correlated with the prognosis of COAD, KIRC, LUAD, and THYM, where low expression of *PARM1* in COAD, KIRC, and LUAD predicted poor patient prognosis (**Figure 8B**).

## 4 Discussion

Abnormal gene expression is a vital factor in the development and metastasis of osteosarcoma, and the genes that produce abnormal expression in this complex biological process are necessarily not single, so it is important to analyze and find new molecular markers that affect the progression of osteosarcoma (16, 17). In the past decades of research, the exploration of aberrantly expressed genes in osteosarcoma has never stopped. To find the role of ferroptosis death-related genes in the development of osteosarcoma, Ding et al. (18) classified osteosarcoma by the method of non-negative matrix decomposition clustering and found significant differences in prognosis and immune status between two subgroups, and found that ferroptosis death-related genes could influence the immune status of the tumor microenvironment, thus affecting the development and prognosis of osteosarcoma. In addition, lipid metabolism genes were used to cluster osteosarcoma patients into two subgroups by consensus, and it was found that the expression of lipid metabolism genes correlated with the immune microenvironment and could be used to accurately predict the osteosarcoma prognosis (19).

In this study, we first compared the differential genes between normal tissues and osteosarcoma tissues in the GSE16088 set, and found 5005 cases of differential genes. In order to further explore the impact of differential genes on the survival of osteosarcoma. We performed WGCNA analysis on 34 patients in the GSE16091 data set to screen the genes related to the survival of osteosarcoma patients. The results were intersected with the differential genes and found that a total of 9 genes were highly correlated with the survival of osteosarcoma. Then the prognostic model was constructed by LASSO and Survival analysis identified *PARM1* as a favorable factor for osteosarcoma patients. In order to further verify the accuracy of our model construction, we built a prognostic risk model in the target database, and further tested that low expression of *PARM1* indicates poor prognosis of osteosarcoma patients, which further verified the accuracy of our model.

In addition, cellular experiments were performed to verify *PARM1* was lowly expressed in the osteosarcoma cell line saos-2. Studies have shown that *PARM1* is considered a new potential oncogene (20). Prostate androgen-regulated mucin-like protein 1 is an integral secreted protein that accumulates mainly in the Golgi apparatus as well as in early and late endosomes, and *PARM-1* increases ERK1/2, AKT, and STAT3 phosphorylation. In colorectal cancer, *PARM1* may be its potential novel prognostic biomarker (21). In prostate cancer cells, *PARM1* ectopic expression increased cell proliferation (22). In addition, *PARM1* expression is stimulated by pro-inflammatory cytokines and is associated with endoplasmic reticulum stress (23).

We performed a pan-cancer expression and prognostic analysis of *PARM1* and found that *PARM1* showed a trend of low expression in most cancers, and low expression of *PARM1* in COAD, KIRC, and LUAD predicted poor prognosis of patients, which also confirmed *PARM1* as a poor prognostic factor. In conclusion, in our study, we identified *PARM1* as a poor prognostic factor in osteosarcoma by bioinformatics based on the GEO database, and low expression of *PARM1* predicted poor prognosis in osteosarcoma patients. Although we verified the heterogeneous expression of *PARM1* in normal tissues and osteosarcoma cells in cellular experiments, to further explore the mechanism of action of *PARM1* affecting the prognosis of osteosarcoma patients.

## Data availability statement

The datasets presented in this study can be found in online repositories. The names of the repository/repositories and accession number(s) can be found in the article/**Supplementary Material**.

## Author contributions

HF: Writing the article, data collection, conception and design. LW: Conception and design, analysis and interpretation, critical revision of the article. JL: Writing the article, critical revision of the article, methodology. SW: Formal analysis, original draft, critical revision of the article. All authors contributed to the article and approved the submitted version.
