# Supplementary figures and images for: The bioinformatic approach identifies PARM1 as a new potential prognostic factor in osteosarcoma
Source: Front Oncol. 2023 Mar 6;12:1059547. doi: 10.3389/fonc.2022.1059547 (PMC10025378; doi:10.3389/fonc.2022.1059547)

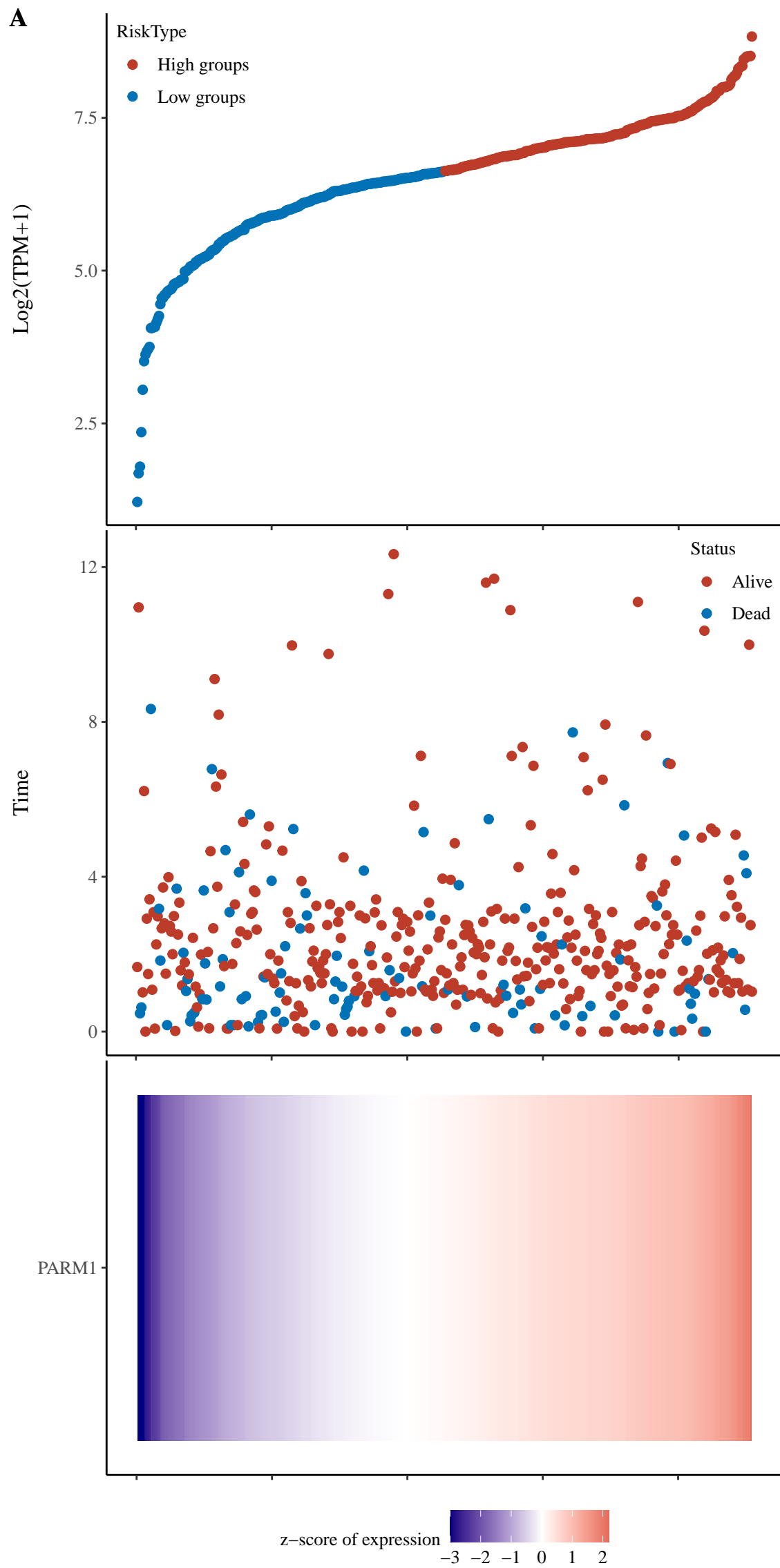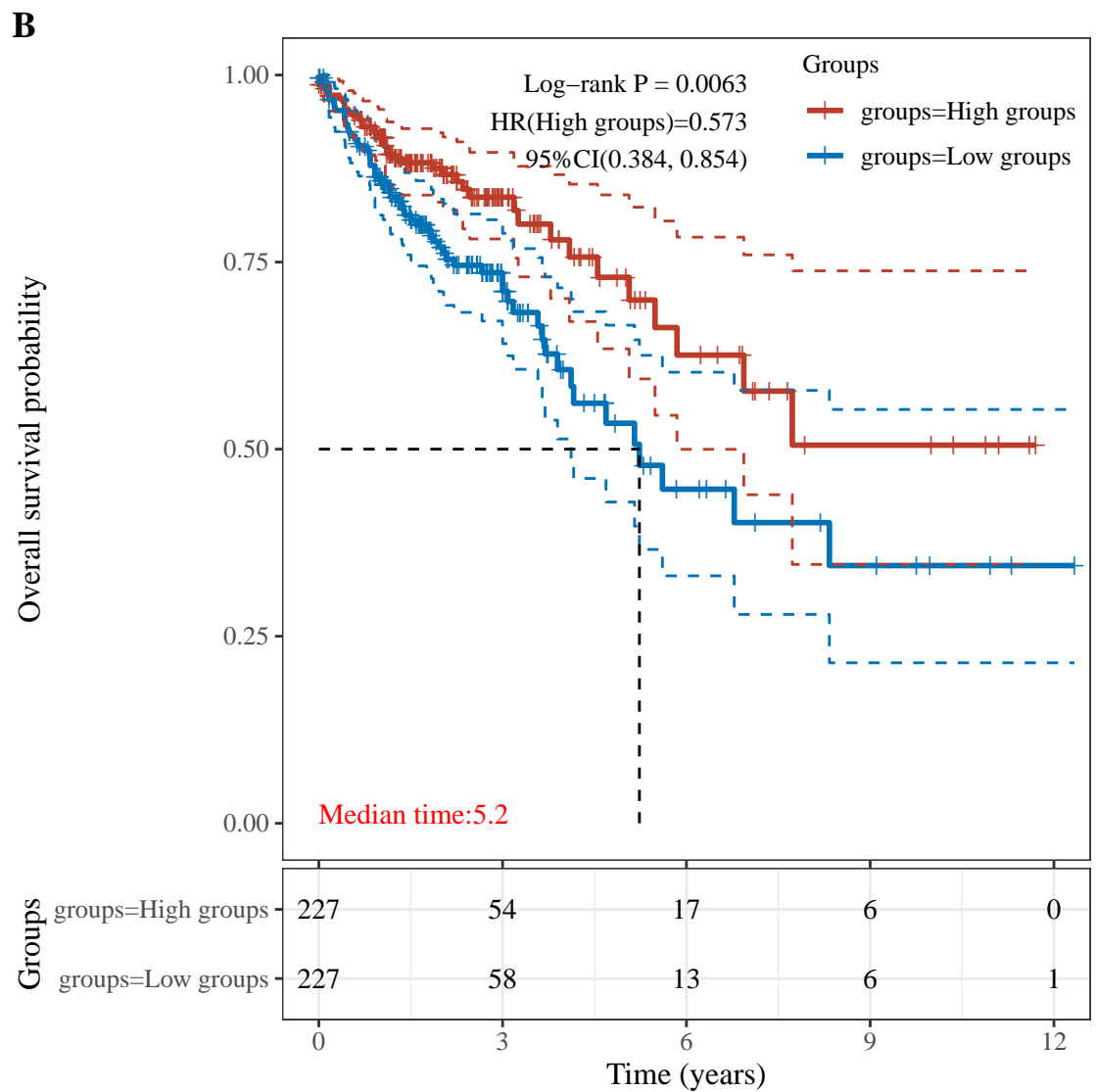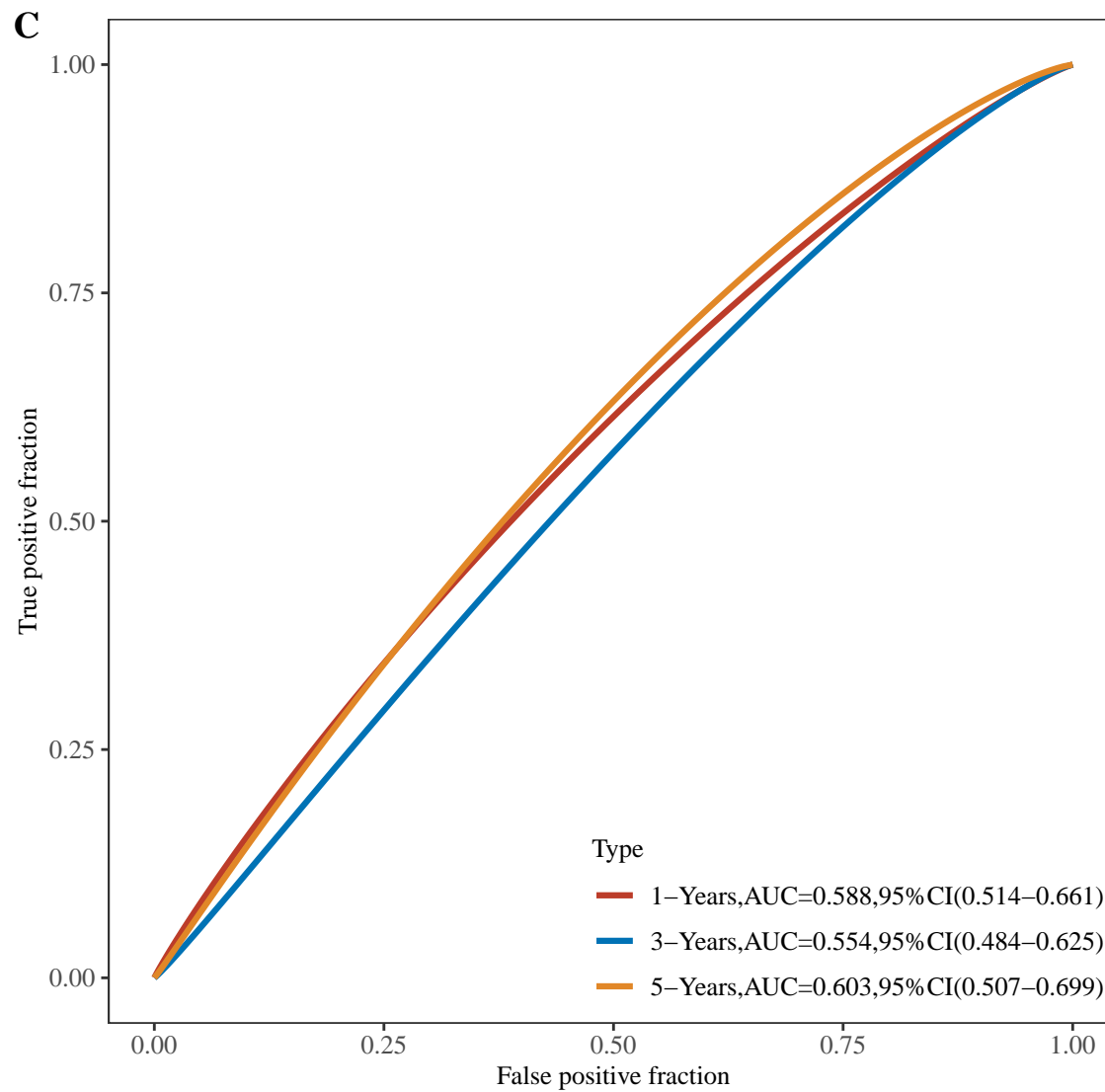

Supplement: Supplementary file 1 [file DataSheet_1.zip › data/pancancer/pancancer/COAD.pdf]

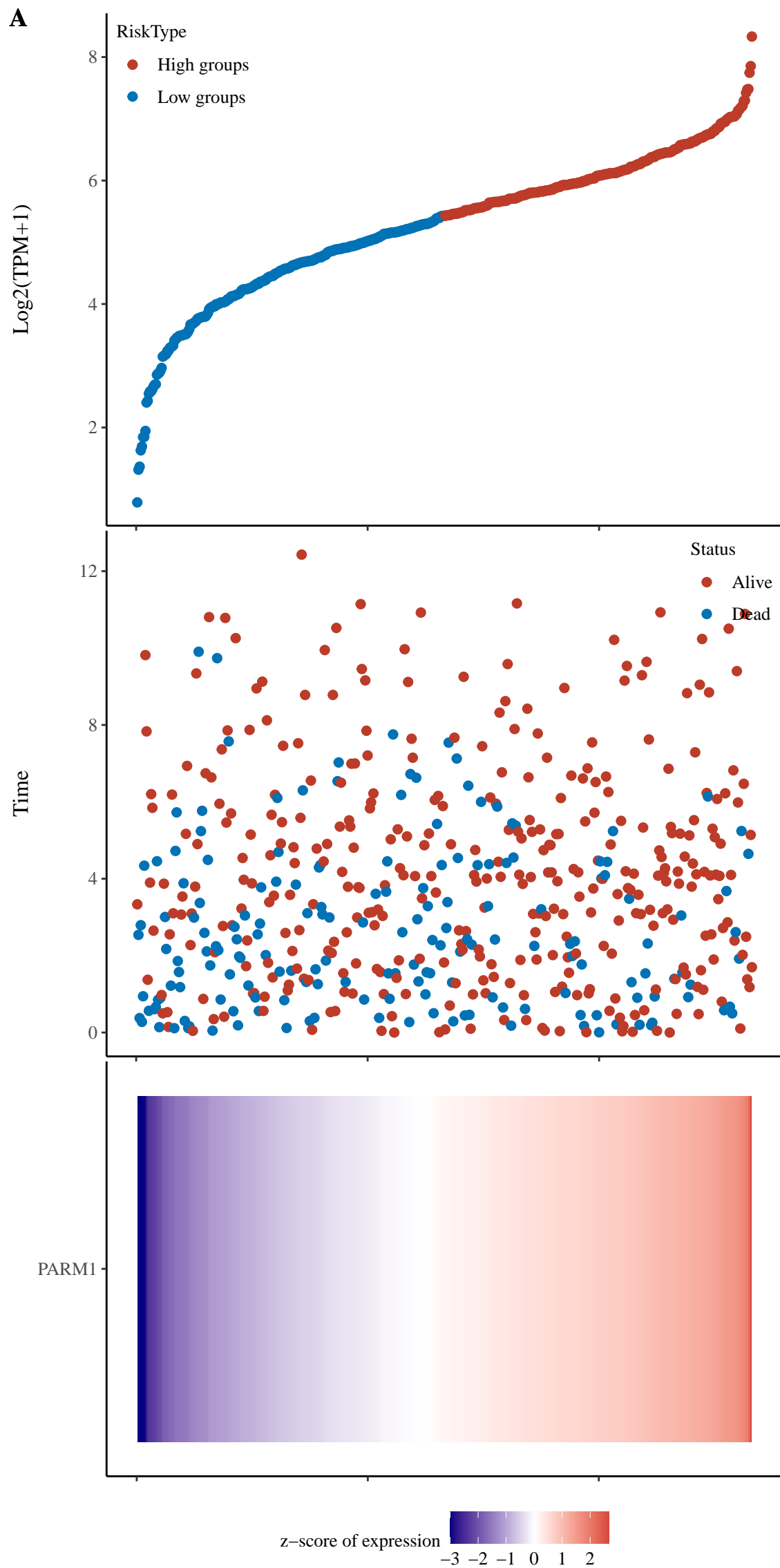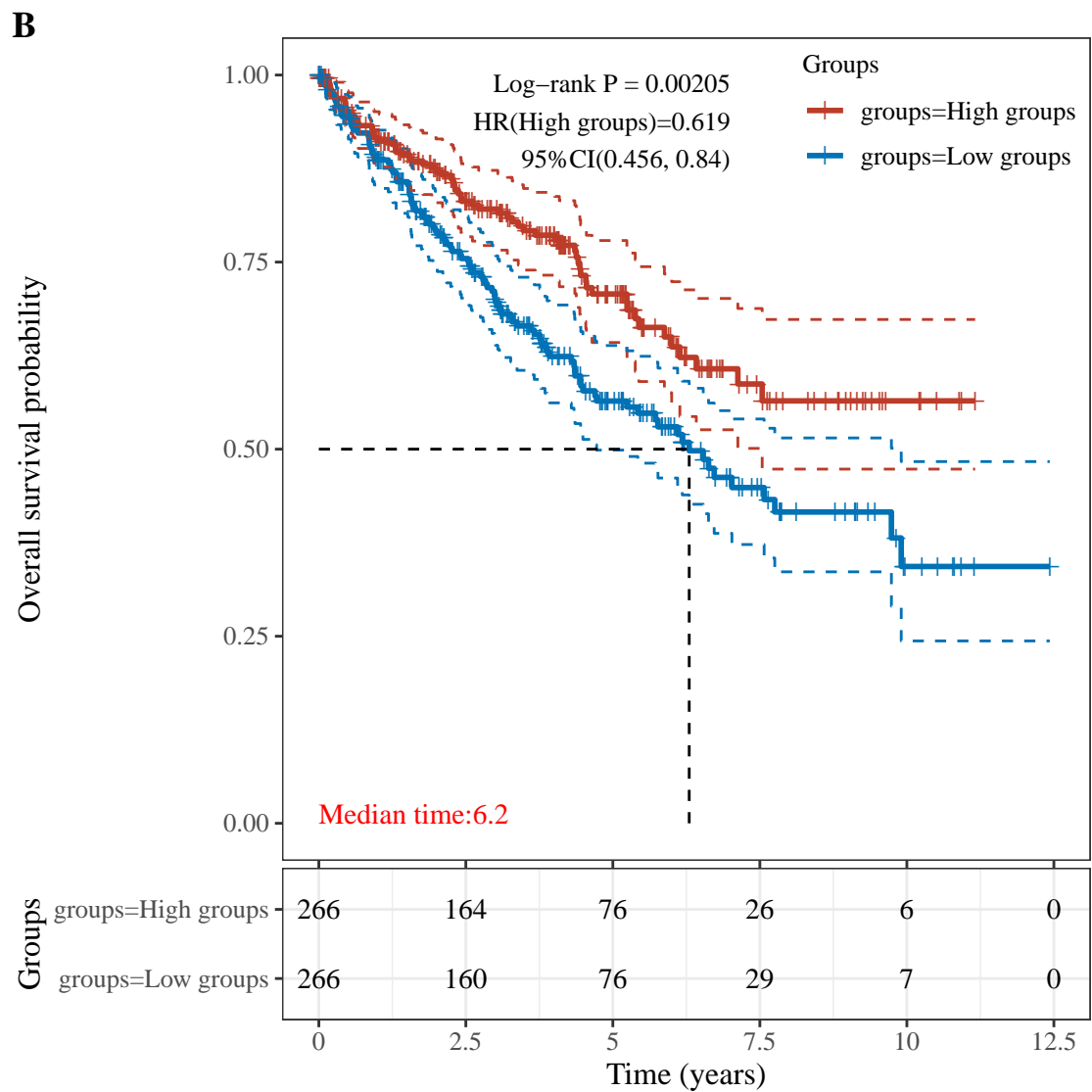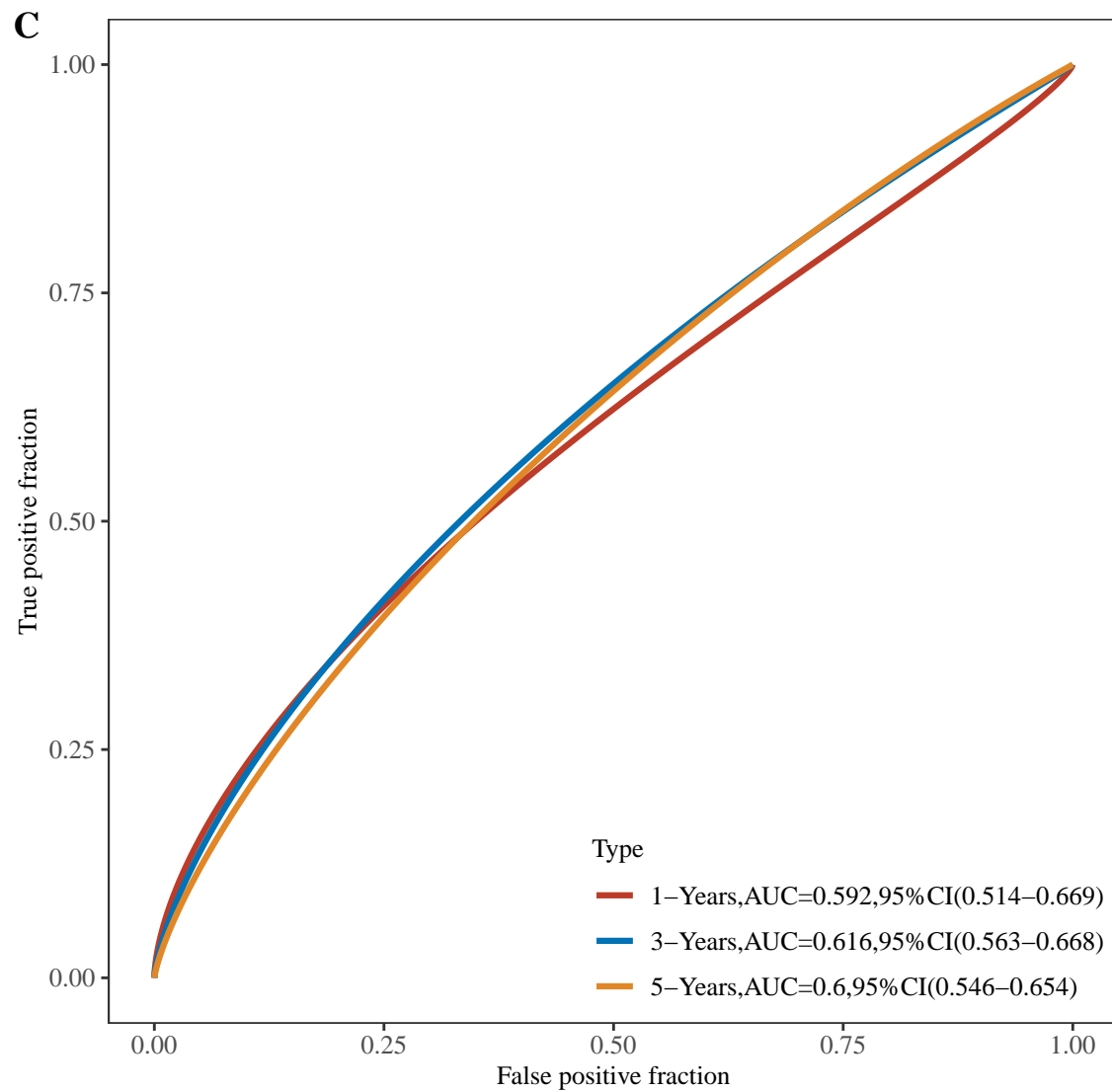

Supplement: Supplementary file 1 [file DataSheet_1.zip › data/pancancer/pancancer/KIRC.pdf]

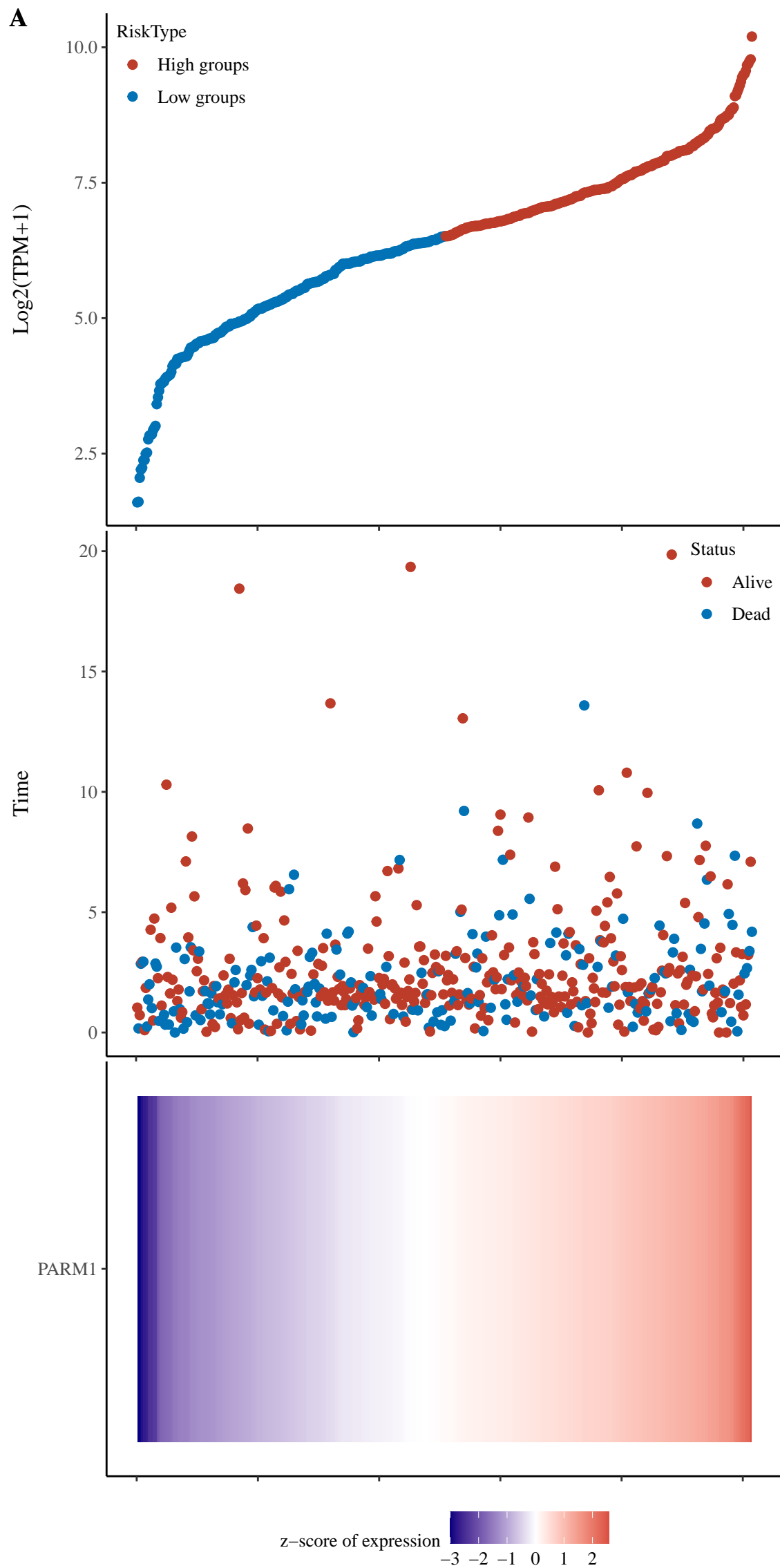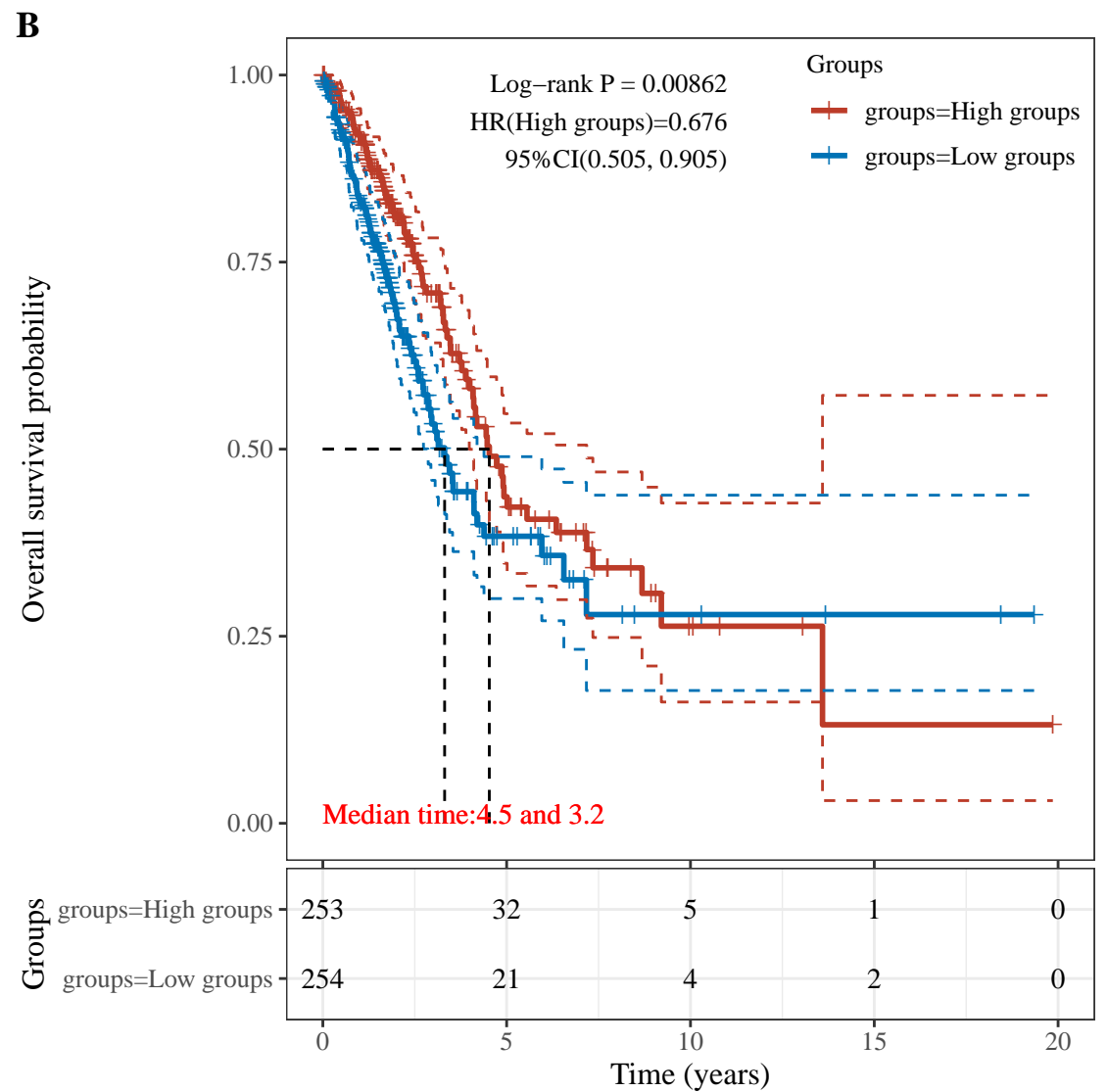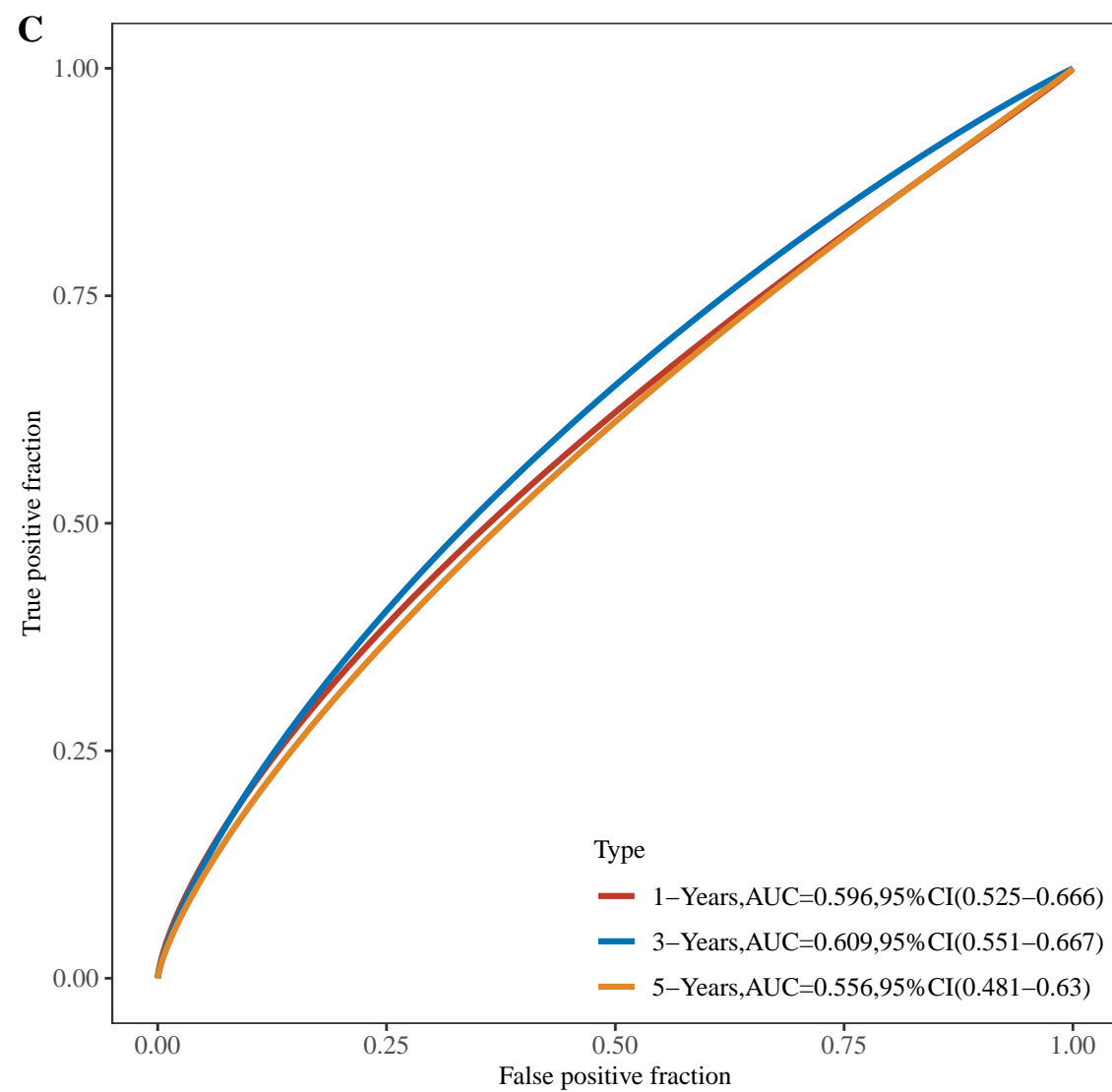

Supplement: Supplementary file 1 [file DataSheet_1.zip › data/pancancer/pancancer/LUAD.pdf]

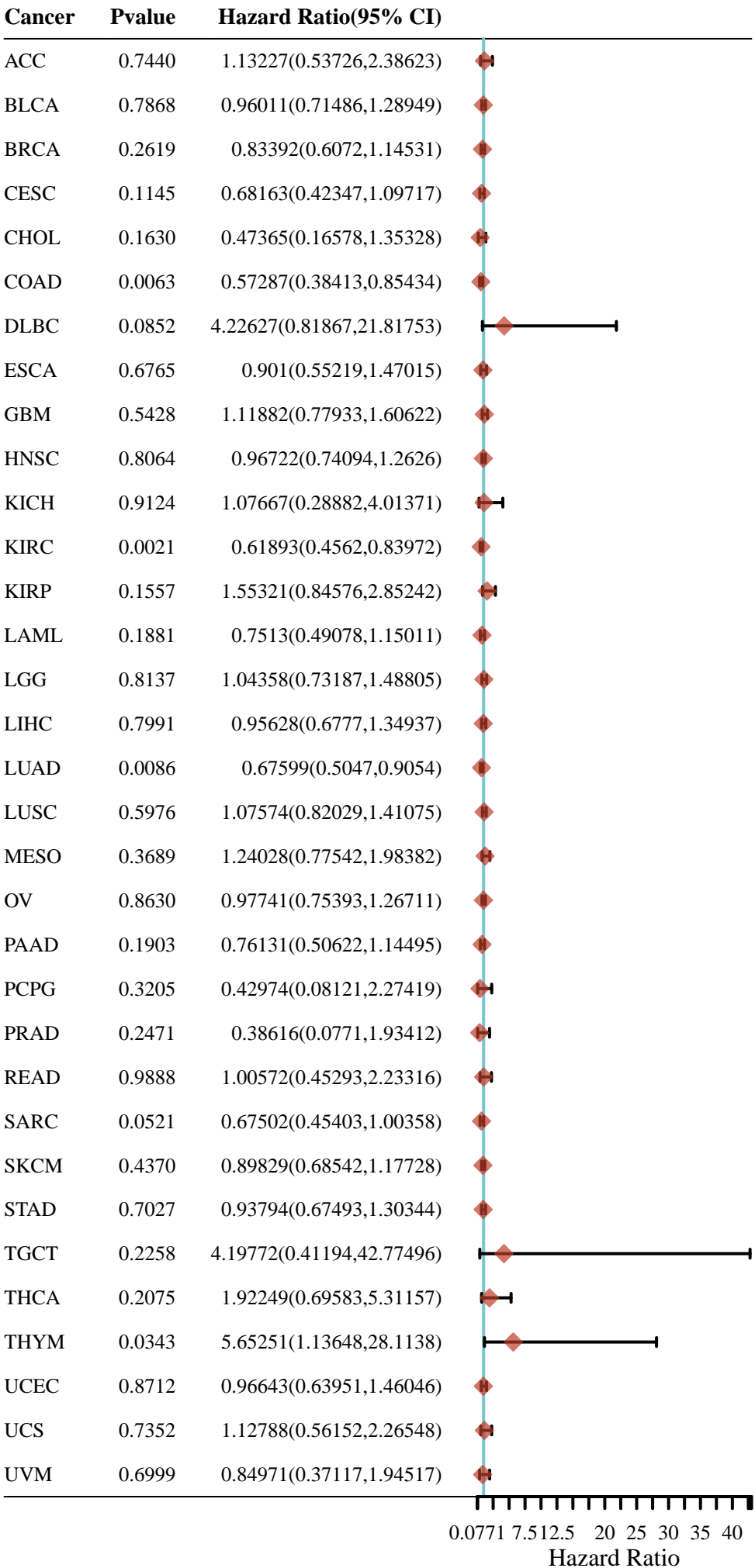

Supplement: Supplementary file 1 [file DataSheet_1.zip › data/pancancer/pancancer/Pan-cancer prognosis/forestplot_Fig.pdf]

The expression of PARM1  
Log<sub>2</sub> (FPKM+1)

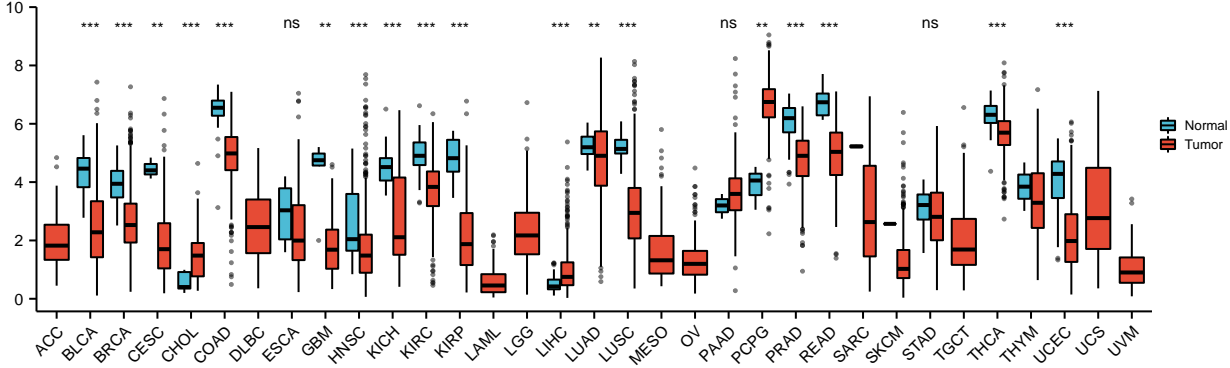

Supplement: Supplementary file 1 [file DataSheet_1.zip › data/pancancer/pancancer/PARM1.pdf]

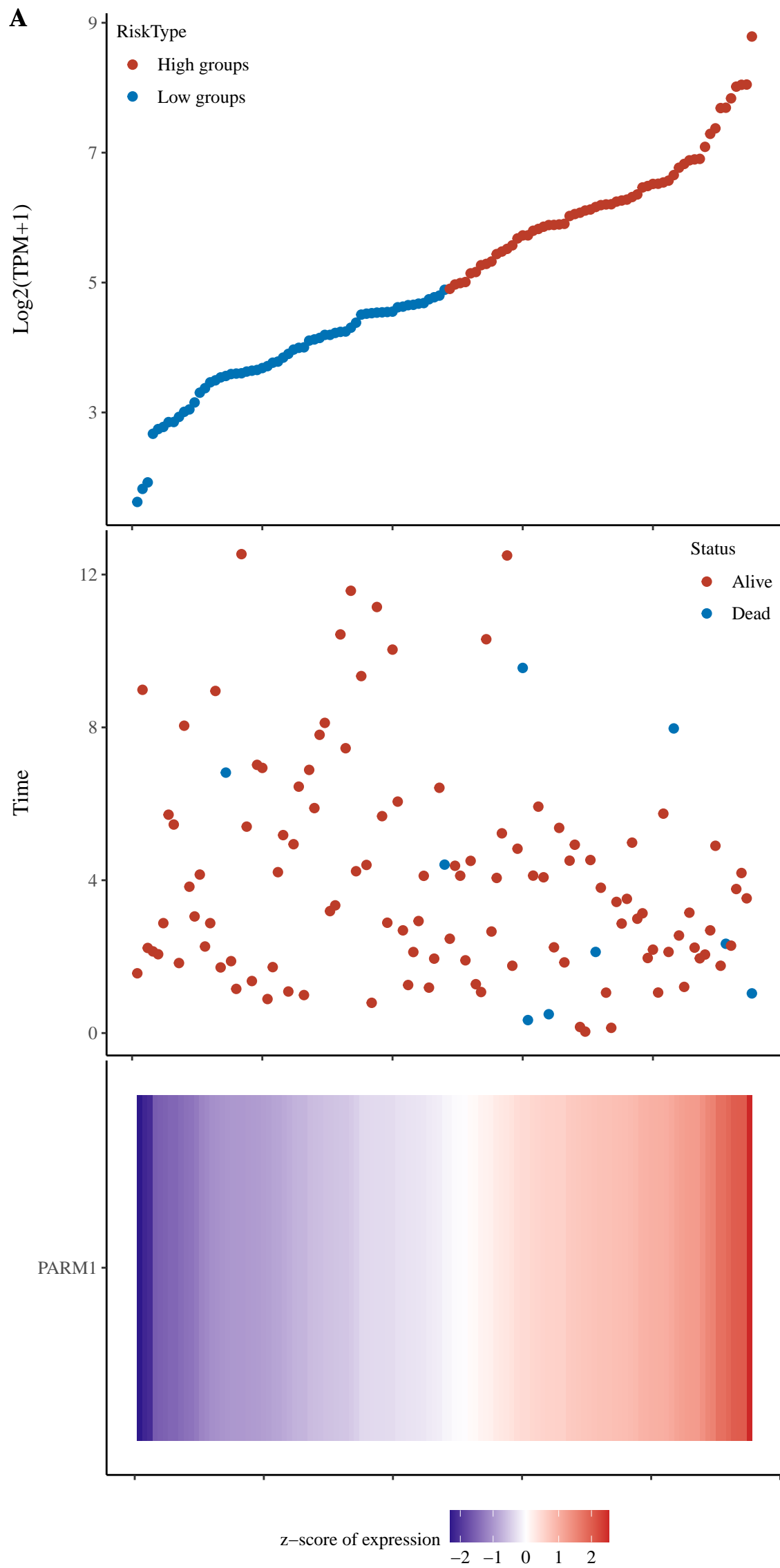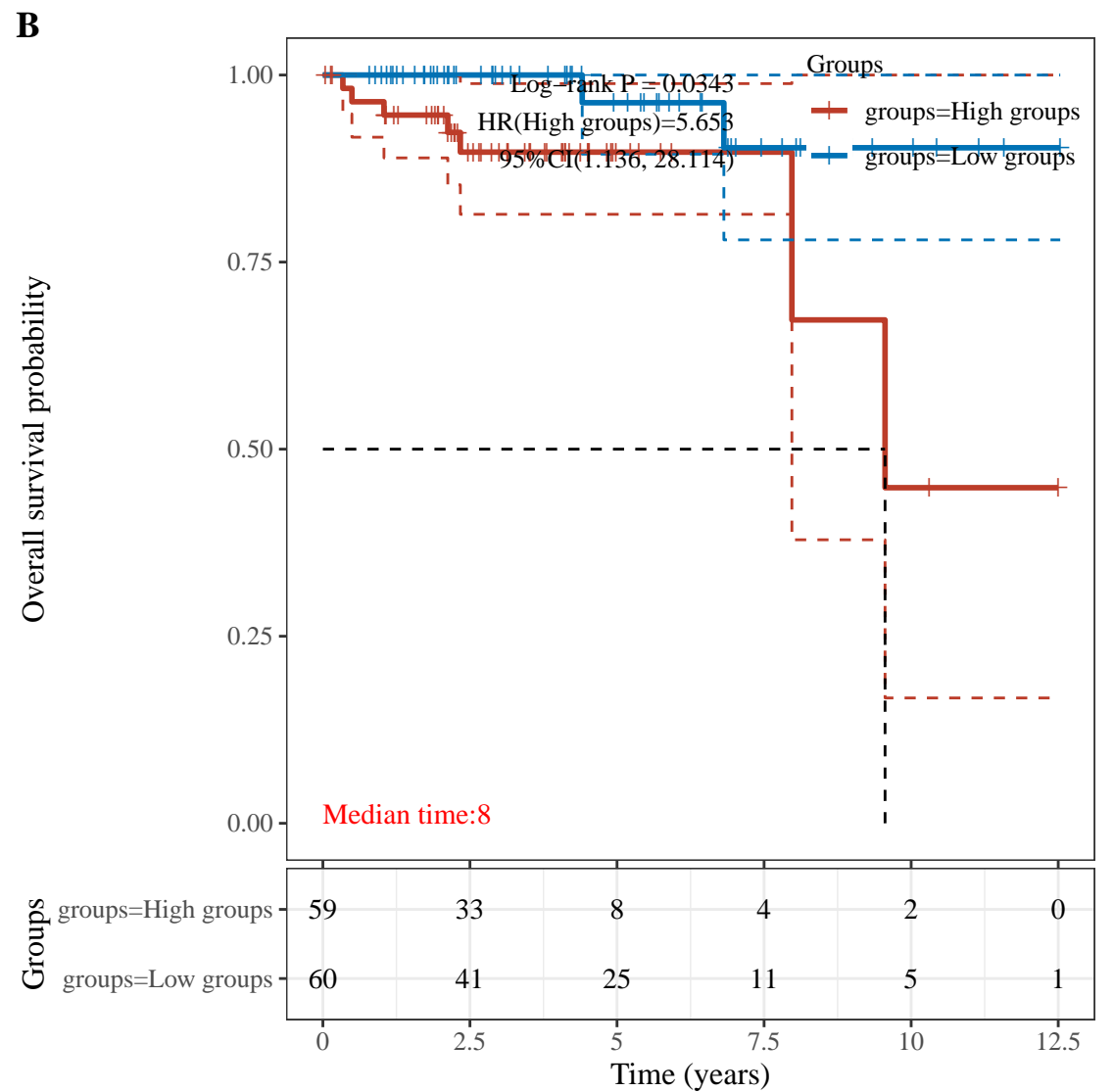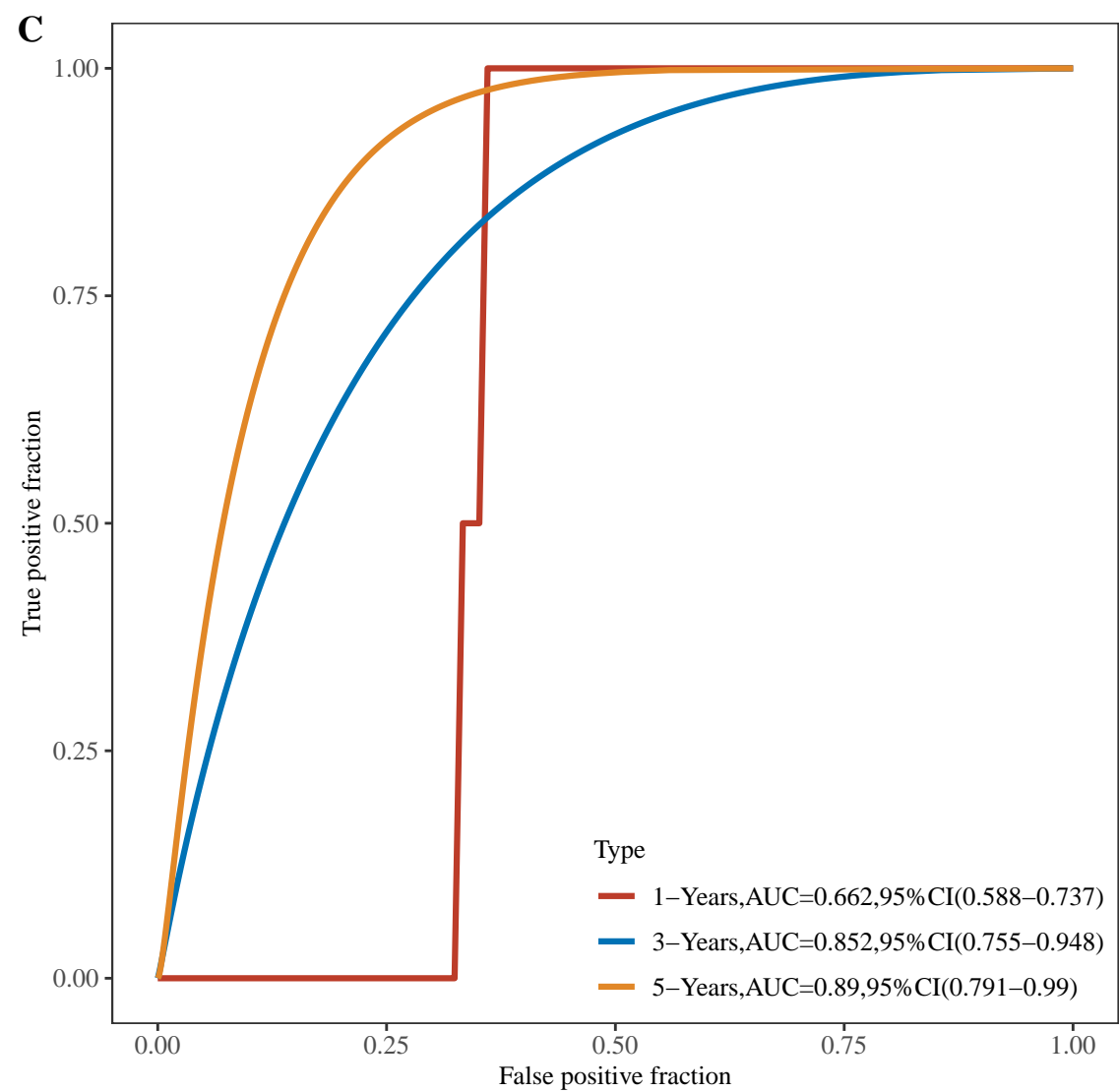

Supplement: Supplementary file 1 [file DataSheet_1.zip › data/pancancer/pancancer/THYM.pdf]

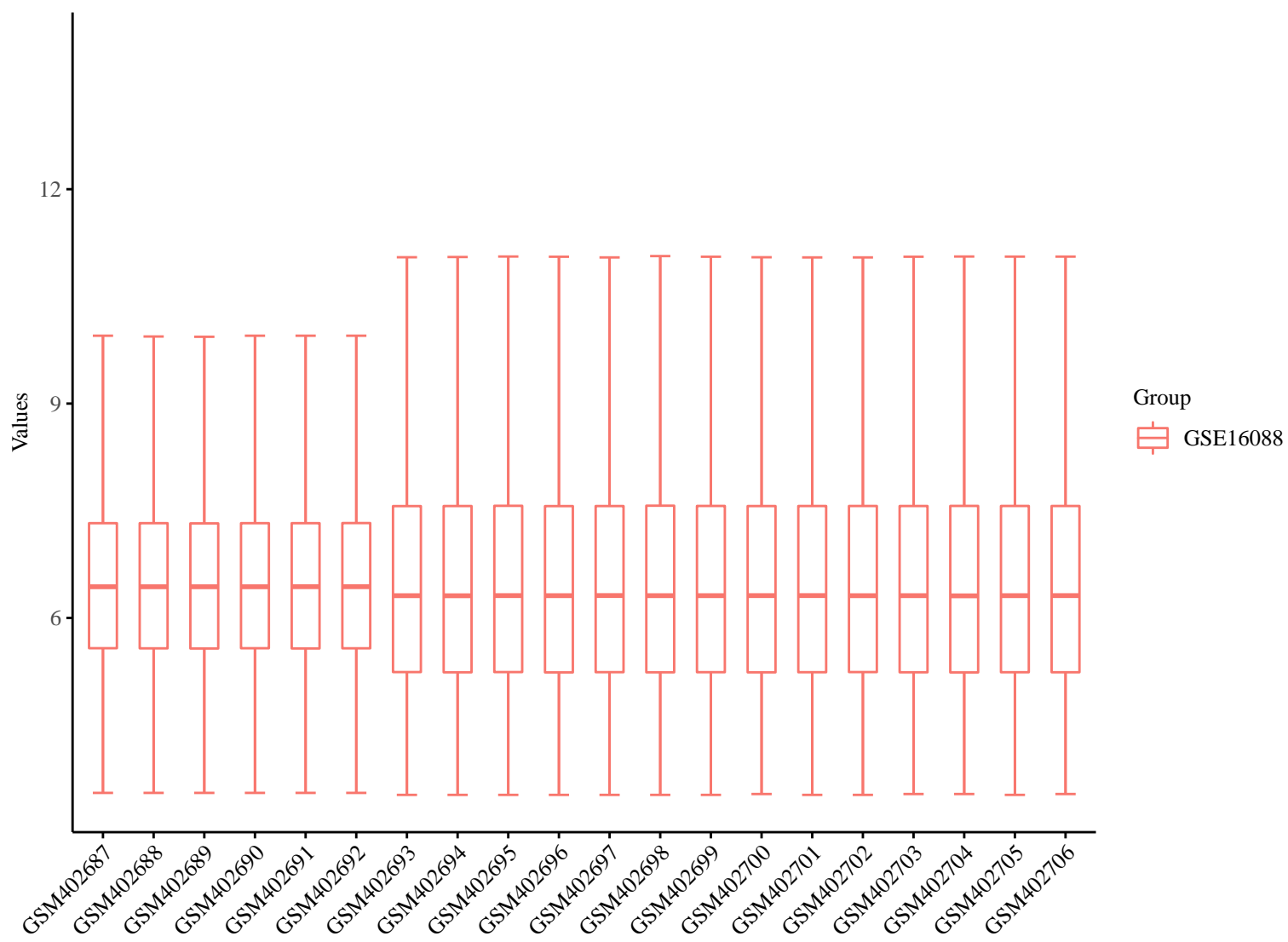

**D** wilcox.tests p=0.00098

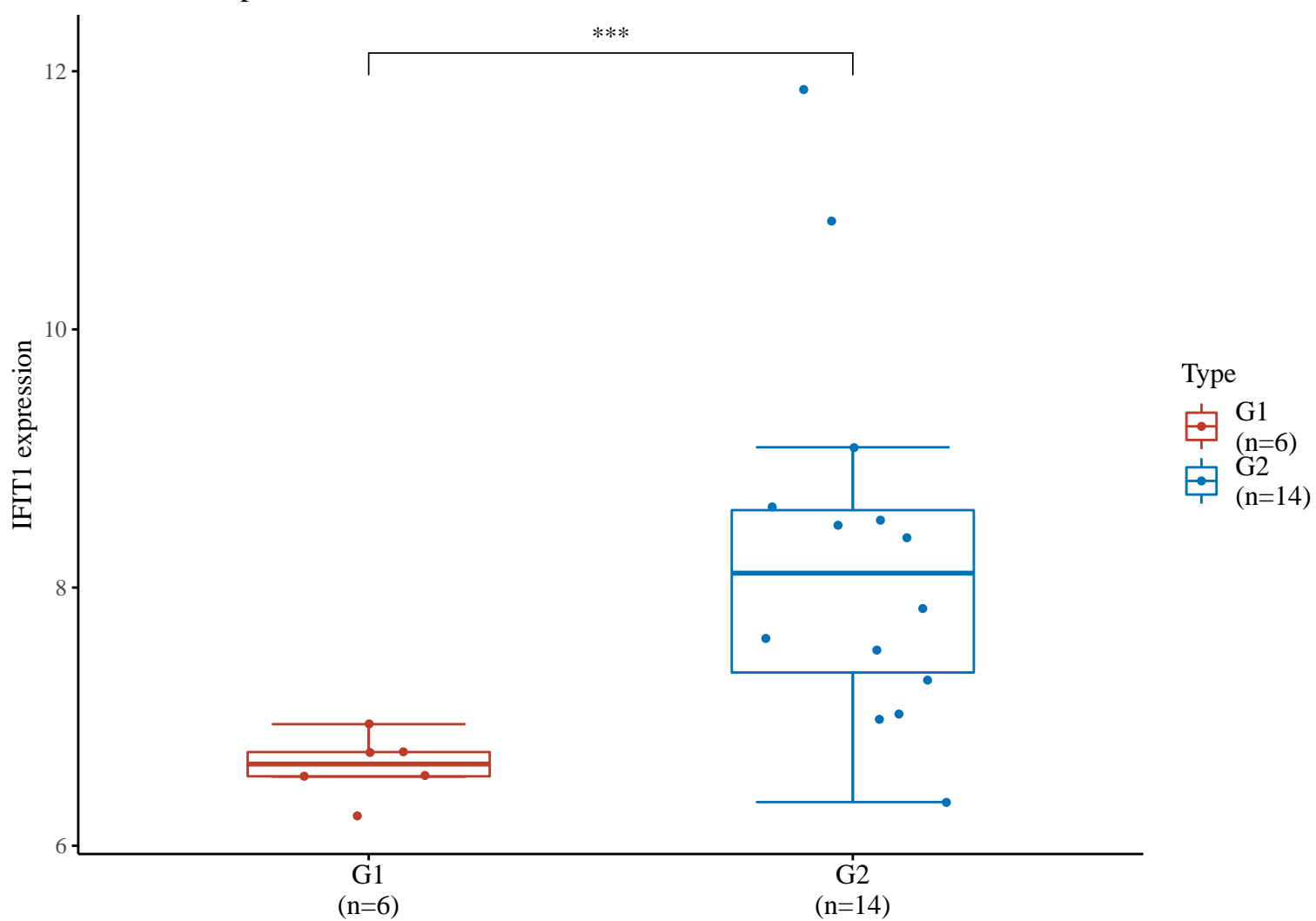

Supplement: Supplementary file 1 [file DataSheet_1.zip › data/Single gene prognosis/Single gene expression/IFIT1.pdf]

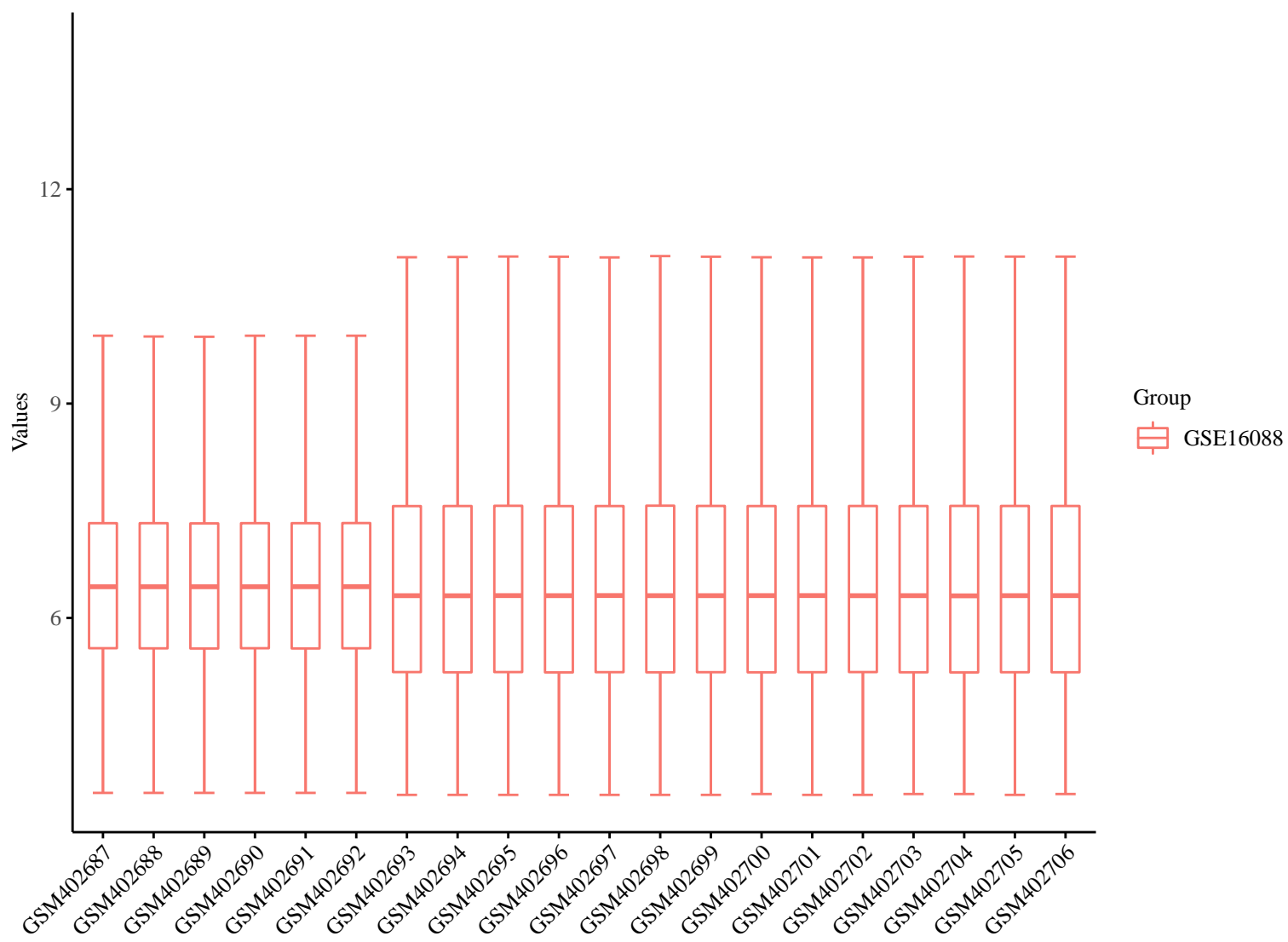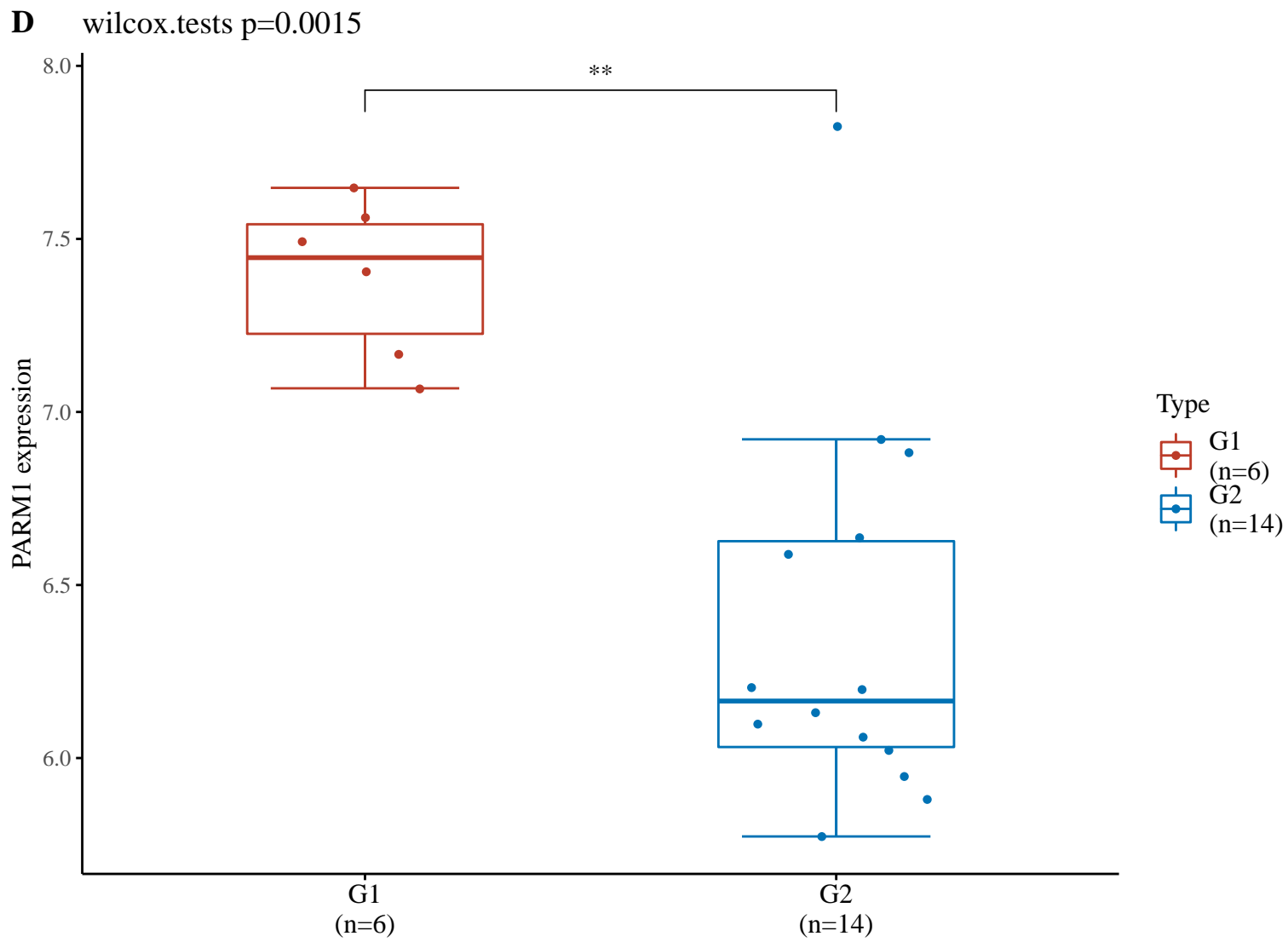

Supplement: Supplementary file 1 [file DataSheet_1.zip › data/Single gene prognosis/Single gene expression/PARM1.pdf]
